# Determinant Factors of Stress in Caregivers of Patients With Schizophrenia: Cross-Sectional Study

**DOI:** 10.2196/70648

**Published:** 2025-07-03

**Authors:** Isymiarni Syarif, Hasnawati Amqam, Saidah Syamsuddin, Veni Hadju, Syamsiar Russeng, Yusran Amir

**Affiliations:** 1 Department of Public Health, Faculty of Public Health, Hasanuddin University, Jl. Perintis Kemerdekaan Km. 10 Tamalanrea, Makassar, 90245, Indonesia, 62 8114630476; 2 Department of Psychiatry, Faculty of Medicine, Hasanuddin University, Makassar, Indonesia; 3 Department of Nutrition, Faculty of Public Health, Hasanuddin University, Makassar, Indonesia; 4 Department of Occupational Safety and Health, Faculty of Public Health, Hasanuddin University, Makassar, Indonesia; 5 Department of Health Administration and Policy, Faculty of Public Health, Hasanuddin University, Makassar, Indonesia

**Keywords:** caregiver, stress caregiver, schizophrenia, determinant factors, cross-sectional study

## Abstract

**Background:**

Caregivers of individuals with schizophrenia face ongoing psychological and emotional burdens due to the chronic and relapsing nature of the disorder and the complexity of caregiving. Prolonged exposure to caregiving stress characterized by emotional exhaustion, role overload, and lack of social support has been consistently associated with poor mental health outcomes among caregivers, including depression and anxiety.

**Objective:**

This study aimed to assess stress levels among caregivers of patients with schizophrenia and identify the key determinants of caregiver stress.

**Methods:**

This study used a cross-sectional survey that was conducted between June and August 2024 at the Labakkang District Health Center, South Sulawesi, Indonesia. A total of 110 female caregivers participated in the study. Data were collected using validated questionnaires to measure stress levels and related factors. Statistical analyses included chi-square tests to identify associations and partial least squares structural equation modeling to examine the strength and direction of relationships between variables.

**Results:**

This study included 110 female caregivers of individuals with schizophrenia. The majority were early older people (48/110, 44%), had a basic level of education (elementary to junior high school; 45/110, 46%), were unemployed (83/110, 75%), and had been providing care for more than 10 years (42/110, 38%). A total of 58 of 110 (53%) caregivers experienced mild levels of stress, while 63 of 110 (57%) caregivers reported a moderate caregiving burden. Additionally, 64 of 110 (58%) caregivers reported challenges related to patient treatment nonadherence, and 58 of 110 (53%) caregivers experienced low levels of social stigma. Most caregivers (69/110, 63%) adopted adaptive coping strategies; however, more than half reported low levels of knowledge (59/110, 54%) and limited access to health information (73/110, 66%). The chi-square analysis identified several statistically significant associations with stress: age (*P*=.03), education (*P*<.001), caregiving burden (*P*<.001), knowledge (*P*<.001), coping strategies (*P*<.001), treatment nonadherence (*P*=.004), and perceived stigma (*P*=.003). Further, partial least squares structural equation modeling analysis showed that caregiving burden (*r*=0.672), stigma (*r*=0.921), and limited knowledge (*r*=0.909) were positively correlated with stress. In contrast, social support was strongly negatively associated with stress (*r*=−0.872), indicating its protective role.

**Conclusions:**

These findings underscore the critical need for targeted interventions that enhance social support networks, reduce stigma, and strengthen caregivers’ coping capacities. Strengthening these dimensions is essential to mitigating the psychological toll of caregiving and sustaining caregivers’ functional well-being. Evidence increasingly supports that empowering caregivers through structured support systems and educational initiatives can substantially alleviate stress-related burdens and improve care continuity for individuals with schizophrenia.

## Introduction

The role of caregivers in supporting individuals with mental disorders, such as schizophrenia, is crucial, particularly in assisting patients with medication adherence, providing emotional support, and helping with daily living activities. However, this often results in significant physical, emotional, and social burdens [1]. These challenges are exacerbated when caregivers are confronted with unpredictable psychotic symptoms, limited knowledge about their condition, and social stigma from the surrounding community [2,3].

Several studies have indicated that caregivers of individuals with schizophrenia experience moderate to high levels of stress, which directly impacts their psychological well-being, manifesting in symptoms such as anxiety, depression, and chronic fatigue [4,5]. This emotional burden not only affects caregivers’ quality of life but also diminishes the effectiveness of patient care, potentially leading to higher relapse rates, increased hospitalization, and additional strain on mental health systems [6,7].

In Indonesia, the situation is even more complex owing to limited access to mental health information, pervasive societal stigma, and a lack of structured social support systems [8,9]. Many caregivers struggle to fully understand a patient’s condition and often feel inadequately equipped or unsupported in providing optimal care. Moreover, there are few community-based or primary care interventions specifically designed to help caregivers manage their psychosocial burdens. This highlights the urgent need for evidence-based approaches that are practically applicable in local contexts and primary health care settings.

The psychosocial stressors faced by caregivers are highly complex and interrelated, encompassing factors such as low mental health literacy, prolonged caregiving duration, caregiver’s psychological state, subjective burden, patient nonadherence to treatment, and insufficient family or social support [10,11]. Therefore, a comprehensive psychosocial approach is required. Previous research has demonstrated that strong social support, improved mental health literacy, and adaptive coping skills are protective factors against caregiver stress [12].

Nonetheless, most existing studies have addressed emotional and social burdens in isolation without considering the dynamic interplay among multiple psychosocial factors [13]. Furthermore, the application of advanced statistical techniques, such as partial least squares structural equation modeling (PLS-SEM), to study caregiver stress remains limited, especially in evolving countries like Indonesia. This method holds significant promise for mapping complex latent relationships among variables, such as stress, stigma, knowledge, subjective burden, social support, and trust [14,15]. Such studies are essential for advancing the development of robust evidence-based interventions [16].

Therefore, this study aimed to holistically identify and analyze the psychosocial factors influencing stress among caregivers of individuals with schizophrenia in Indonesia using a cross-sectional design and PLS-SEM approach. These findings are expected to offer meaningful empirical contributions to the field of community mental health and serve as a foundation for developing more contextual, humane, and effective interventions to enhance caregiver well-being and patient quality of life [17].

## Methods

### Design, Setting, and Participants

This study used a cross-sectional survey design to assess stress levels and identify the key determinant factors among caregivers of individuals with schizophrenia. A cross-sectional web-based survey was conducted with adults (aged 18 y and older) living with a mental health disorder. The self-reported survey was administered on the web-based survey platform Qualtrics. The survey is described according to the CHERRIES (Checklist for Reporting Results of Internet E-Surveys) [18]. The study was conducted within the jurisdiction of 3 community health centers in Labakkang Subdistrict, Pangkep Regency, South Sulawesi, Indonesia. At the time of the survey, there were 138 registered caregivers of individuals diagnosed with schizophrenia, of whom 110 were female. These caregivers were distributed across the 3 participating community health centers.

The participants were selected through purposive sampling. The sample size was determined using the Roscoe formula [19], which states that for multivariate analysis, the sample size should be at least 10 times the number of variables (*R*=n×<10), where *n* is the number of variables. Given that the study involved 11 variables, a minimum of 110 participants was deemed necessary.

Purposive sampling was used to recruit caregivers who met the following inclusion criteria: (1) registered as caregivers for a person diagnosed with schizophrenia at one of the selected health centers, (2) aged 18 years or older, and (3) actively involved in daily care activities. Caregivers who were unable to participate because of illness, language barriers, or unwillingness to provide consent were excluded. The selected sample represented diverse demographic and caregiving backgrounds, contributing to the generalizability of the findings to the target population.

Data collection was conducted over three months: 28 caregiver responses were collected in June, 55 in July, and 27 in August. These data were used to assess caregivers’ stress levels and identify contributing personal, situational, and environmental factors.

### Data Collection

The survey was conducted between June and August 2024, through direct home visits to caregivers of individuals with schizophrenia. To maximize response rates and ensure data quality, trained public health enumerators were engaged in the survey in collaboration with mental health officers from participating health centers. The fieldwork was coordinated by local government authorities and the Pangkep District Health Office. Community health volunteers (cadres) also supported field activities by facilitating access to and encouraging community participation.

Data were collected using a structured questionnaire that included questions regarding emotional burden, care demands, social support, and caregiver stress levels. Stress levels were evaluated using the National Alliance for Caregiving Questionnaire in collaboration with the American Association of Retired Person [20]. Stigma was measured using the Internalized Stigma of Mental Illness scale [21]. Caregivers’ coping mechanisms were assessed using the Coping Orientation to Problems Experienced Inventory and McMaster Family Assessment Device [22,23], and social support was evaluated using the Multidimensional Perceived Social Support questionnaire [24]. The burden of caregiving was quantified using Zarit’s Caregiver Burden [25], and caregiver knowledge was measured through the Knowledge Assessment Schizophrenia Test [26,27]. Additionally, medication noncompliance among patients with schizophrenia was assessed using the Medication Compliance Report Scale [28]. Each instrument was rigorously validated at the Bungoro Health Center, Pangkep Regency, to ensure high reliability and validity (Table 1).

**Table 1.** Validity and reliability of assessment tools determinant factors of stress level on caregivers of patients with schizophrenia in Pangkep Regency, Indonesia, 2024.

Assessment tools	Reliability
National Alliance for Caregiving Questionnaire in collaboration with AARP^a^	0.982^b^
Internalized Stigma of Mental Illness scale	0.9^c^
The COPE^d^ Inventory and the McMaster Family Assessment Device	0.989^c^
Multidimensional Perceived Social Support	0.952^b^
Zarit’s Caregiver Burden	0.989^c^
Knowledge Assessment Schizophrenia Test	0.966^c^
Medication Compliance Report Scale	0.949^c^

a AARP: American Association of Retired Persons.

b *P*<.01.

c *P*<.001.

d COPE: Coping Orientation to Problems Experienced.

### Data Statistical Analysis

Manual thematic coding was conducted to categorize data into predefined themes, such as stress levels and determinant factors, including patient characteristics, caregiver burden, stigma, knowledge, coping strategies, access to health information, medication nonadherence, social support, and caregiving duration. The coding framework was informed by the existing literature and refined during the initial data familiarization. The coded data were then tabulated using Excel (Microsoft Corp) and further analyzed using SPSS (version 29; IBM Corp) for both descriptive and inferential statistics.

**Figure 1.**
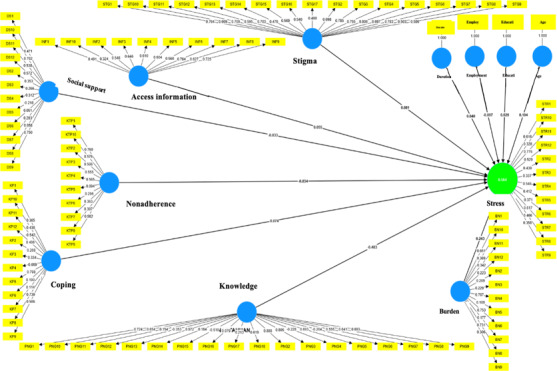
PLS model specification diagram (first estimation) determinant factors of stress level on caregiver of patients with schizophrenia in Pangkep Regency, Indonesia, 2024. PLS: partial least squares.

To examine the relationship between exogenous and endogenous variables, the data were analyzed using PLS-SEM with SmartPLS (version 4; SmartPLS GmbH) [29-32]. The evaluation process consists of two stages: the measurement model and the structural model [32,33]. The PLS-SEM was selected for this study for several reasons. First, it is well-suited for advancing existing theories [34]. Second, PLS-SEM is highly effective for analyzing complex models, especially in exploratory research. Third, it allows for the analysis of the entire model as a cohesive entity, rather than isolating individual components [35]. Finally, PLS-SEM enables the simultaneous evaluation of both structural and measurement models, ensuring more robust and precise results [36]. The exogenous variables included personal, situational, and social environmental determinants, while the endogenous variables included stress levels. The proposed model is shown in the supplement in Multimedia Appendix 1 and Figures1  2. This study quantitatively analyzed the determinants of caregiver stress in schizophrenia care, focusing on caregiver coping, burden of care, stigma, medication nonadherence, and social support. Chi-square tests were used to examine the interrelationships among the measurable variables.

**Figure 2.**
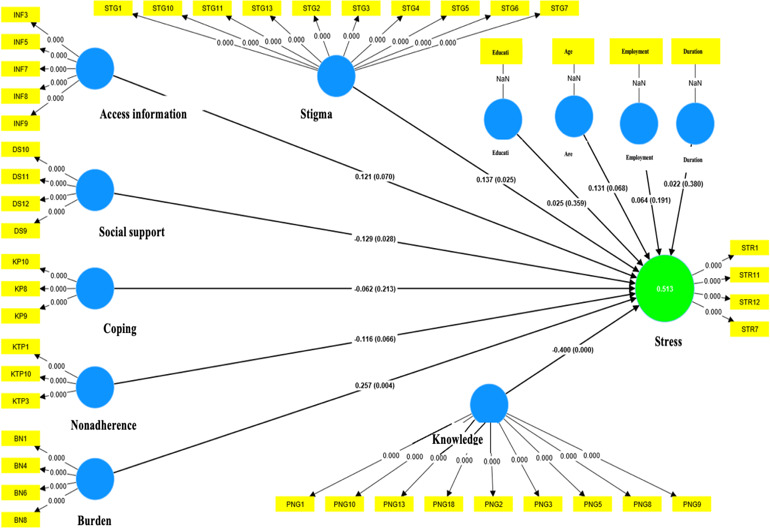
Outer model diagram of PLS respecification (second estimation) determinant factors of stress level on caregiver of patients with schizophrenia in Pangkep Regency, Indonesia, 2024. PLS: partial least squares.

### Ethical Considerations

Ethical approval was obtained from the Faculty of Public Health Ethics Committee, Hasanuddin University (25240930270). Written informed consent was obtained from all participants before data collection. The participants were assured of their right to withdraw at any time, and strict confidentiality was maintained by anonymizing and securely storing all data. Nonmonetary incentives were provided to acknowledge the participants’ time and contribution, as the study involved minimal risk.

## Results

### Stress Levels of Caregivers

Caregivers of individuals with schizophrenia often experience elevated stress levels owing to the complex emotional, physical, and social demands associated with caregiving. These stressors can substantially affect psychological well-being and social functioning. As shown in Table 2, a majority of caregivers (58/110, 53%) reported experiencing mild stress, while 6 out of 110 (5%) caregivers reported severe stress. These findings suggest that caregiving for individuals with schizophrenia imposes considerable psychological pressure, even though extreme stress levels are not prevalent among most respondents. According to Lazarus and Folkman’s stress and coping theory, stress arises when individuals perceive that their environmental demands exceed their available coping resources. In this context, although most caregivers did not exhibit signs of severe stress, the predominance of mild stress nonetheless indicated a persistent psychological strain embedded in the caregiving experience.

**Table 2.** Distribution of stress levels of caregivers of patients with schizophrenia in Pangkep Regency, Indonesia.

Stress levels	Value, n (%)
Normal	46 (42)
Mild stress	58 (53)
Severe stress	6 (5)
Total	110 (100)

From a practical perspective, these findings underscore the need for structured support systems at the community and primary health care levels. Early intervention to manage mild stress is essential to prevent escalation to severe stress, particularly considering the long-term burden of caring for individuals with schizophrenia. Primary care centers and mental health services can play an active role by providing education, stress management training, and access to caregiver support networks. This may include the promotion of mobile health interventions as digital platforms for ongoing monitoring and caregiver education.

### Personal Determinant Factors

Understanding the personal determinant factors that influence caregivers is crucial for addressing their stress and challenges. The analysis categorizes these determinants into 4 key aspects: age, education, employment, and care duration. Each of these factors plays a significant role in shaping the caregiving experience and the associated psychological burden.

The majority of caregivers (48/110, 44%) were early older individuals (Table 3). Of the 110 caregivers, 37 (34%) were late adults. Age influences both physical and psychological capacities to manage caregiving-related stress [37]. Early older caregivers may benefit from greater life experiences, but may also begin to face declining energy levels. In practice, this highlights the need for tailored attention to the physical capacity and emotional support of older caregivers to help them sustain their caregiving roles without excessive fatigue.

**Table 3.** Distribution of personal determinant factors of patients with schizophrenia in Pangkep Regency, Indonesia, 2024.

Personal determinant factors	Value, n (%)
Age	
Early adult	25 (22)
Late adult	37 (34)
Early older individuals	48 (44)
Education	
Without school	23 (21)
Elementary school	50 (45)
Senior high school	33 (30)
Higher school	4 (4)
Employment	
Unemployed	83 (75)
Employed	27 (25)
Duration of care (years)	
Less than 1 to 4	31 (28)
5 to 10	37 (34)
Over 10	42 (38)

The highest proportion of caregivers had only completed elementary school (50/110, 45%), whereas 23 of 110 (21%) caregivers had no formal education (Table 3). Lower educational attainment may affect their ability to access health care information, cope with caregiving stress, and seek professional assistance when required. Individuals with lower educational backgrounds often face difficulties in accessing, processing, and understanding essential information for caring for people with mental illnesses, which may exacerbate caregivers’ emotional burdens [38]. From a practical standpoint, simple, visual-based educational tools, particularly those delivered through technology-based interventions, may help reduce caregiver stress by providing easy-to-understand information and enhancing caregivers’ capacity to care for individuals with schizophrenia.

A striking 83 out of 110 (75%) caregivers were unemployed, whereas only 25% (27/110) were employed (Table 3). This high rate of unemployment among caregivers may intensify stress levels, as the inability to work or maintain stable employment often leads to feelings of being trapped in the caregiving role without adequate financial or social support [39]. In practical terms, economic assistance or skill training programs that enable caregivers to pursue flexible employment opportunities could help alleviate both financial and emotional burdens. The inability to balance work and caregiving responsibilities may also contribute to burnout and reduce overall quality of life.

The duration of caregiving varied: 42 out of 110 (38%) caregivers provided care for over 10 years, followed by 37 out of 110 (34%) caregivers for 5 to 10 years (Table 3). The duration of caregiving may affect caregivers’ stress levels. According to the stress theory, the longer an individual serves as a caregiver, the greater the potential for emotional and physical exhaustion, commonly referred to as burnout [38,40]. In practical terms, interventions that provide continuous support, such as coping training and psychosocial assistance, are crucial for reducing long-term caregiver stress and helping caregivers manage the challenges associated with prolonged caregiving.

### Situational Determinant Factors

Caregiving for individuals with schizophrenia presents significant challenges, often leading to psychological, emotional, and physical burdens for caregivers. Situational determinant factors play a crucial role in understanding how external conditions influence caregivers’ well-being and ability to provide adequate care. These factors include caregiver burden, coping mechanisms, and knowledge of schizophrenia, all of which have direct implications for caregivers’ quality of care and overall mental health.

Based on Table 4, it can be concluded that the burden experienced by caregivers of patients with schizophrenia was significant. The majority (63/110, 57%) of caregivers reported experiencing moderate burden, while 33 out of 110 (30%) caregivers reported a heavy burden reflecting the ongoing pressure resulting from complex caregiving demands. These findings are consistent with the Transactional Model of Stress and Coping, which posits that stress arises when demands exceed an individual’s available resources [41]. In practical terms, this highlights the need for interventions that not only focus on stress management but also strengthen social support, education, and technological tools such as mobile health to help caregivers cope with challenges adaptively.

**Table 4.** Distribution of situational determinant factors of patients with schizophrenia in Pangkep Regency, Indonesia, 2024.

Situational determinant factors	Value, n (%)
Caregiver burden	
Minimal burden	14 (13)
Moderate burden	63 (57)
Heavy burden	33 (30)
Coping caregiver	
Maladaptive coping	41 (37)
Adaptive coping	69 (63)
Knowledge	
Low knowledge	59 (54)
Adequate knowledge	51 (46)
Medication nonadherence	
High nonadherence	46 (42)
Low nonadherence	64 (58)

Table 4 shows the coping mechanisms, indicating that 69 out of 110 (63%) caregivers applied adaptive coping, while 41 out of 110 (37%) caregivers still used maladaptive coping. Based on the Transactional Model of Stress and Coping, these findings suggest that the majority of caregivers are able to adapt to stress constructively, but a significant proportion still rely on maladaptive coping, highlighting the need for targeted interventions [40,41]. Interventions such as coping skills training and social support are effective in enhancing adaptive coping and reducing caregiver burden.

Table 4 shows the level of knowledge about schizophrenia. The data indicated that 59 of 110 (54%) caregivers had low knowledge, while 46% (51/110) possessed adequate knowledge. Low knowledge may influence caregivers’ perceptions of risk, benefits of actions, and self-efficacy in caring for individuals with schizophrenia [42]. Practically, these findings highlight the importance of structured educational interventions, such as digital psychoeducation or community-based training, to enhance caregivers’ mental health literacy. Nurfurqoni et al [8] demonstrated that digital psychoeducational interventions significantly improve caregivers’ knowledge and skills, which in turn, positively impact burden reduction and coping improvement.

Table 4 shows that another factor influencing caregiver well-being is their level of medication nonadherence. The data indicated that 46 of 110 (42%) caregivers had high medication nonadherence. The finding that 42% (46/110) of caregivers reported high medication nonadherence highlights a critical risk factor for schizophrenia management. According to the Health Belief Model, nonadherence may result from perceived barriers or low perceived benefits of consistent medication use [42]. Practically, this underscores the need for targeted interventions that address caregiver knowledge, beliefs, and routines, such as mobile-based reminders, psychoeducation, and caregiver-inclusive treatment planning, to improve adherence and prevent relapse.

### Social Environment Determinant Factors

The social environment plays a crucial role in determining stress levels among caregivers of patients with schizophrenia, where low social support, societal stigma, and limited access to health information contribute to an increased psychological burden.

**Figure 3.**
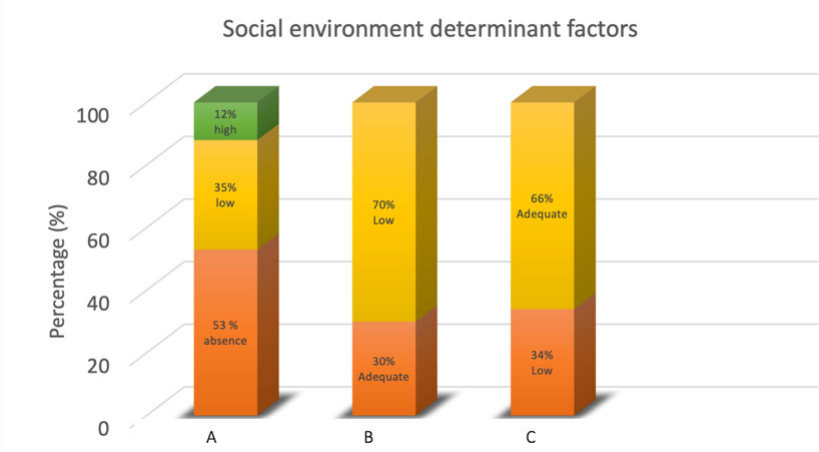
Distribution of social environment determinant factors—(A) stigma; (B) social support; and (C) access to health information—of patients with schizophrenia in Pangkep Regency, Indonesia 2024.

Figure 3A shows that the stigma related to schizophrenia is a significant challenge for caregivers. The data revealed 2 categories of stigma: low stigma, with 58 of 110 (53%) caregivers and high stigma, with 13 of 110 (12%) caregivers. Internalized stigma can negatively affect caregivers’ mental health, self-efficacy, and willingness to seek support [43]. Practically, these findings highlight the need for community-based interventions such as peer support groups and antistigma education to reduce negative perceptions and promote caregiver resilience, particularly for those still experiencing high stigma.

Figure 3B shows that social support is a crucial factor in reducing stress and enhancing caregivers’ well-being. The data indicated that 30 of 110 (33%) caregivers experienced low social support, while 70 of 110 (77%) caregivers received adequate support. Social support functions as a resource that helps individuals cope with stress, serving as both an emotional and practical buffer for caregivers caring for individuals with schizophrenia [44]. Adequate social support can reduce burnout and improve caregivers’ quality of life, which, in turn, enhances the quality of patient care. Conversely, lack of social support increases vulnerability to stress, consistent with Social Support Theory, which suggests that caregivers who feel isolated are more susceptible to emotional stress and difficulties in caring for patients [45]. These findings highlight the importance of community-based interventions to strengthen social support networks and improve access to mental health services to reduce caregiver burden and enhance the quality of care provided to patients.

Figure 3C shows that access to health information played a crucial role in determining the effectiveness of caregivers’ support for patients. The data revealed that 73 of 110 (66%) caregivers had limited access to health information, while only 37 of 110 (34%) caregivers had sufficient access. Limited access to health information can hinder decision-making and increase stress, consistent with Health Information Access Theory [46]. Practically, such limitations can reduce the quality of care provided to patients with schizophrenia. Therefore, interventions such as digital education and health literacy programs are essential to strengthen caregivers’ capacities.

### Significant Determinant Factors of Caregiver Stress

Caregiving for individuals with schizophrenia presents significant challenges that often lead to elevated stress levels. Understanding the key determinants of caregiver stress is essential for developing targeted interventions to enhance well-being. The results indicated that certain factors were significantly related to caregiver stress (*P*<.05).

The chi-square analysis identified several factors that were significantly associated with caregiver stress. Regarding personal determinants, the findings revealed that age was significantly associated with caregiver stress (*P*=.03), with younger caregivers tending to experience higher stress levels than older caregivers(Table 5). The educational level also demonstrated a significant relationship with stress levels (*P*<.001), with caregivers with lower educational attainment experiencing higher stress than those with secondary or higher education levels. In contrast, employment status did not show a statistically significant relationship with caregiver stress (*P*=.05), although employment may provide positive distractions and emotional support. Similarly, caregiving duration did not exhibit a significant association with caregiver stress (*P*=.07), suggesting that caregiving experience beyond 10 years does not necessarily alleviate or exacerbate stress.

**Table 5.** Determinant factors of stress level on caregivers of patients with schizophrenia in Pangkep Regency, Indonesia, cross-sectional study, 2024.

Personal determinant factors	Stress level						Total, n (%)		*P* value^a^
	Normal, n (%)		Mild stress, n (%)		Severe stress, n (%)				
Age									.03^a^
Early adult	8 (7.3)		15 (13.7)		2 (1.8)		25 (22.7)		
Late adult	16 (14.5)		19 (17.3)		2 (1.8)		37 (33.6)		
Early older individual	22 (20)		24 (21.8)		2 (1.8)		48 (43.7)		
Education									<.001
Without school	12 (10.9)		11 (10)		0 (0)		23 (20.9)		
Elementary school	28 (25.5)		10 (9.1)		12 (10.9)		50 (45.5)		
Senior high school	6 (5.5)		26 (23.7)		1 (0.9)		33 (30)		
Higher school	0 (0)		4 (3.6)		0 (0)		4 (3.6)		
Employment									.05
Employed	16 (14.5)		10 (9.1)		1 (0.9)		27 (24.5)		
Unemployed	30 (27.3)		48 (43.6)		5 (4.5)		83 (75.5)		
Duration of caregiving (years)									.07
<1-4	18 (16.4)		13 (11.7)		0 (0)		31 (28.2)		
5-10	15 (13.6)		21 (19.1)		1 (0.9)		37 (33.6)		
10+	13 (11.8)		24 (16.4)		5 (4.5)		42 (38.2)		
Burden of caregiver									<.001
Minimal	13 (11.8)		1 (0.9)		0 (0)		14 (12.7)		
Moderate burden	32 (29.1)		25 (24.5)		4 (3.6)		63 (57.3)		
High burden	1 (0.9)		30 (27.2)		2 (1.8)		33 (30)		
Coping of caregiver									<.001
Adaptive	10 (9.1)		54 (49.1)		6 (5.5)		69 (62.7)		
Maladaptive	36 (32.7)		4 (3.6)		1 (0.9)		41 (37.3)		
Knowledge									<.001
Adequate	45 (40.9)		6 (5.4)		0 (0)		51 (46.7)		
Low	1 (0.9)		36 (32.7)		6 (5.5)		59 (53.6)		
Medication noncompliance									.004^a^
High	11 (10)		34 (31)		1 (0.9)		46 (41.8)		
Low	35 (31.8)		24 (21.9)		5 (4.5)		64 (58.2)		
Stigma									.003^a^
Absence	12 (10.9)		26 (23.7)		1 (0.9)		39 (35.5)		
Low	34 (30.9)		29 (26.3)		5 (4.5)		70 (64.7)		
High	0 (0)		3 (2.7)		0 (0)		3 (2.7)		
Social support of caregiver									.26
Adequate	34 (30.9)		38 (34.5)		5 (4.5)		77 (70)		
Low	12 (10.9)		20 (18.1)		1 (0.9)		33 (30)		
Access to health information									.25
Adequate	13 (11.8)		23 (20.9)		1 (0.9)		37 (33.6)		
Low	33 (30)		35 (31.9)		5 (4.5)		73 (66.4)		

a Chi-square test (significance set at *P*<.05).

Regarding situational determinants**,** the results indicated that caregiving burden was significantly associated with caregiver stress (*P*<.001), where a higher burden was correlated with increased stress (Table 5) Caregivers’ coping also showed a significant relationship with stress (*P*<.001), with caregivers using maladaptive coping strategies experiencing higher stress levels than those using adaptive coping mechanisms. Furthermore, medication noncompliance by the care recipient was significantly related to caregiver stress (*P*=.004).

Regarding social environmental determinants, the findings revealed that social stigma was significantly associated with caregiver stress (*P*=.003), with caregivers experiencing high stigma being more vulnerable to moderate to severe stress than those experiencing low stigma (Table 5). Social support did not show a significant relationship with caregiver stress (*P*=.26), although caregivers with limited social support reported higher stress levels than those with sufficient support (Table 5). Similarly, access to health information was not significantly associated with stress levels (*P*=.25), although caregivers with inadequate access to health-related information reported increased anxiety and uncertainty, which may have indirectly contributed to stress.

These findings underscore that the factors significantly associated with caregiver stress include age, education level, caregiving burden, coping strategies, medication compliance, caregiver knowledge, and social stigma (Table 5). In contrast, employment status, caregiving duration, social support, and access to health information were not significantly associated with caregiver stress (Table 5). These results highlight the importance of targeted interventions to reduce caregiver stress by enhancing knowledge, promoting adaptive coping strategies, and addressing social stigmas.

### Measurement Model Analysis Using PLS-SEM

The PLS-SEM is an exploratory study with structural model development. When the evaluation results of the research model in the second-order factor do not sufficiently meet the levels of validity, reliability, and convergent validity in measuring the relationship between variables and their measurement dimensions, the PLS model needs to be considered. In the respective PLS model, a direct estimation was conducted between information access, social support, stigma, burden, noncompliance, coping, stress, knowledge, duration of care, occupation, education, and age in relation to increased stress. Thus, the model was revised from a second-order factor to a first-order factor.

### Evaluation of the Respecified PLS Outer Model

Evaluation of the inner model or structural model is related to testing the hypothesis of the influence between previously hypothesized research variables. The first structural model evaluation consists of checking the collinearity between variables with the Inner variance inflated factor measure where the inner variance inflated factor value <5 means there is no multicollinearity. The simplified PLS model is expressed as follows. The PLS model is an approach with first-order factor measurement, in which each dimension or aspect directly influences the stress variable. The first estimation result indicates that some indicators are not valid for measuring their respective dimensions and require adjustments by adding or removing certain indicators. In terms of reliability, most variables had a composite reliability value above 0.60, except for coping, which was less reliable. Furthermore, convergent validity has not yet been achieved as the average variance extracted (AVE) value is below 0.50, necessitating further model refinement.

The initial estimation results of the PLS model specification (Figure 1) show that several indicators are invalid for measuring the dimensions of each variable. These indicators exhibited modest correlations when the same variable dimensions were measured. To obtain a successful PLS model, it is necessary to continually add and delete indicators from the model. The first estimation result in the PLS respecification model shows that there are several indicators that are not valid for measuring each dimension of the variable measurement. The indicators were not strongly correlated when measuring the same variable dimensions. Therefore, it is necessary to repeatedly enter and remove indicators from the model to obtain a valid PLS respecification model.

According to Table 6, the PLS respecification model estimation results show that, according to the composite reliability measure, the two variables have low reliability. For the coping dimension, a Cronbach α value of 0.622 was considered trustworthy; however, a composite reliability score of 0.591<0.60 is regarded as unreliable. These findings highlight the need to eliminate erroneous indicators from the PLS model in order to enhance it from the initial estimation. The AVE value of the PLS respecification model was less than 0.50, making the convergent validity results with the AVE unacceptable for the first estimation. Consequently, either eliminating indicators with low outer loadings or selecting indicators with a high degree of correlation are required to improve the model.

**Table 6.** Reliability level of respecification (first estimation) determinant factors of stress level on caregiver of patients with schizophrenia in Pangkep Regency, Indonesia, cross-sectional study, 2024.

Dimension	Cronbach α^a^	Composite reliability (ρ_a)^b^	Composite reliability (ρ_c)^c^	Note
Access to health information	0.842	0.829	0.845	Reliable
Social support caregiver	0.622	0.677	0.639	Reliable
Stigma	0.918	0.934	0.927	Reliable
Burden caregiver	0.709	0.721	0.712	Reliable
Medication nonadherence	0.677	0.685	0.746	Reliable
Coping caregiver	0.622	0.564	0.591	Nonreliable
Knowledge	0.685	0.916	0.775	Reliable
Stress	0.715	0.757	0.789	Reliable

a α<0.6; nonreliable.

b ρ_a (composite reliability)>0.6.

c A measure of internal consistency in a variable in the structural equation modeling (SEM) measurement model.

In the second estimation (Figure 2), the results showed an improvement in validity, with all indicators having outer loadings greater than 0.60 (Table 7). The strongest correlating indicators were identified for each dimension: DS9 for social support, INF3 for information access, STG5 and STG7 for stigma, and STR11 for stress. Additionally, personal determinant factors, such as education, length of care, employment, and age, also had valid outer loadings. Thus, the refined PLS model demonstrated stronger and more reliable results when the variables studied were measured.

**Table 7.** Reliability level of second estimation on determinant factors of stress level on caregiver of patients with schizophrenia in Pangkep Regency, Indonesia, cross-sectional study, 2024.

Dimension	Cronbach α^a^	Composite reliability (ρ_a)^b^	Composite reliability (ρ_c)^c^	AVE^d^^,e^
Access to health information	0.561	0.705	0.807	0.680
Social support caregiver	0.806	0.872	0.887	0.727
Burden caregiver	0.659	0.672	0.815	0.597
Stigma	0.916	0.921	0.930	0.548
Medication nonadherence	0.775	0.820	0.897	0.813
Coping caregiver	0.737	0.790	0.849	0.655
Knowledge	0.897	0.909	0.918	0.588
Stress	0.710	0.728	0.821	0.537

a α<0.6; nonreliable.

b ρ_a (composite reliability)>0.6.

c ρ_c (composite reliability)>0.6.

d AVE: average variance extracted.

e AVE>0.6.

Figure 2 indicates that all indications are true when the outer loadings are greater than 0.60. DS1, DS2, and DS3 are three reliable indicators that measure social support; their outer loadings ranged from 0.696 to 0.938. Access to the information dimension is measured by two reliable indicators, INF3 and INF6, whose outer loadings range from 0.709 to 0.926. A total of 11 indicators, STG1, STG2, STG3, STG4, STG5, STG6, STG7, STG9, STG10, STG11, and STG13, are valid for assessing the stigma dimension, and their outer loadings range from 0.662 to 0.846. BN1, BN6, and BN8 were reliable indicators of the burden dimension. The outer loading ranged from 0.678 to 0.837. Three reliable indicators that measured the coping dimension were KP5, KP8, and KP9; their outer loadings ranged from 0.732 to 0.907. Among these indicators, KP8 exhibited the highest outer loading. Medication noncompliance can be measured using two reliable indicators, KTP3 and KTP10, which have outer loadings ranging from 0.872 to 0.930. Indicator KTP3 exhibited the highest outer load. A total of 8 reliable indicators with outer loadings ranging from 0.648 to 0.900 were used to measure knowledge dimension: PNG1, PNG2, PNG3, PNG5, PNG8, PNG9, PNG10, and PNG13. The PNG2 and PNG13 indicators exhibited the highest external loading. A total of 4 reliable indicators, STR1, STR7, STR11, and STR12, with outer loadings between 0.655 and 0.855, were used to measure the stress dimension, and have identified STR11 as having the largest outer loading.

According to Table 7, the PLS respecification model estimation results show that, according to the composite reliability measure, the two variables have low reliability. For the coping dimension, a Cronbach α value of 0.622 was considered trustworthy; however, a composite reliability score of 0.591<0.60 is regarded as unreliable. These findings highlight the need to eliminate erroneous indicators from the PLS model in order to enhance it from the initial estimation. The AVE value of the PLS respecification model was less than 0.50, making the convergent validity results with the AVE unacceptable for the first estimation. Consequently, either eliminating indicators with low outer loadings or selecting indicators with a high degree of correlation is required to improve the model.

The second estimation indicated that all measurement dimensions were dependable, with Cronbach α and composite reliability values over 0.60 (reliable), indicating a satisfactory reliability level for the second estimation in the PLS model. However, information access has a reliability value of 0.807>0.60 (reliable) according to the composite reliability measure, but 0.561<0.60 (not reliable) according to Cronbach α. Furthermore, the AVE value, which indicates convergent validity, was valid (>0.50).

To evaluate the predictive power of a PLS-SEM model, it is important to ensure that the model can not only explain the relationships in the sample but also accurately predict new data. In the PLS prediction analysis, a comparison between the root mean square error and mean absolute error using a linear regression model was performed to assess the predictive performance of the model. This approach helps identify potential overfitting and ensures the predictive validity of the model.

According to Table 8 in the PLS model, all indicators of the endogenous variable stress (STR1, STR7, STR11, and STR12) exhibited lower root mean square error and mean absolute error values than those of the linear regression model, as shown in Table 8. The test results show that the PLS model proposed in this study has good predictive power. Consequently, the strong predictive power of the PLS model was validated. This study’s PLS model was deemed acceptable based on an examination of the entire model using R-square, Q-square, standardized root mean squared residual, and PLS Predict. Knowledge, burden, social support, and stigma are the three aspects of this model that impact caregiver stress. Although greater load and noncompliance with medication might increase stress, increased understanding can help alleviate stress. This model suggests that caregiver stress is influenced by four factors: knowledge, burden, social support, and stigma. Increased knowledge can help reduce stress, while a greater burden and noncompliance with treatment can increase stress. With this understanding, interventions aimed at increasing caregiver knowledge and reducing caregiver burden by teaching effective coping techniques, such as relaxation, EFT, spiritual therapy, meditation, and yoga, can help caregivers manage stress.

**Table 8.** PLS^a^ predict factors of stress level on caregivers of patients with schizophrenia in Pangkep Regency, Indonesia, cross-sectional study, 2024.

	Q²predict	PLS-SEM_RMSE^b^^,c^	PLS-SEM_MAE^d^	LM_RMSE	LM_MAE
STR1	0.254	0.871	0.640	1.150	0.900
STR7	0.083	0.844	0.597	1.107	0.823
STR11	0.276	0.779	0.579	0.904	0.732
STR12	0.119	1.041	0.812	1.431	1.103

a PLS: partial least squares.

b SEM: structural equation modeling.

c RMSE: root mean square error.

d MAE: mean absolute error.

## Discussion

### Principal Results

This study found that 53% (58/110) of caregivers of individuals with schizophrenia experienced mild stress, whereas 5% (6/110) reported severe stress, indicating that a subset of caregivers faced significant challenges. The PLS-SEM analysis demonstrated that stress was significantly associated with caregiver burden, social support, stigma, and knowledge. The predictive model showed strong relevance, supporting the use of data-driven approaches for identifying the key psychosocial determinants. These findings highlight the importance of targeted interventions to mitigate caregiver stress and improve the quality of care.

These results support the transactional model of stress proposed by Lazarus and Folkman, emphasizing the interplay between personal and environmental factors in shaping stress responses. From a practical perspective, the findings underscore the need to strengthen caregiver capacity through mental health literacy, stigma reduction, and technology-assisted education and counseling. Interventions such as mobile apps, caregiver training, and support programs may help to reduce caregiver burden and improve outcomes for both caregivers and patients.

Policy makers should consider implementing structured and scalable strategies to support caregivers’ well-being and reduce the risk of relapse in individuals with schizophrenia. The findings of this study provide a robust foundation for crafting targeted interventions that address both the psychological needs of caregivers and the quality of care provided to patients. Such interventions should include mental health literacy programs, caregiver training initiatives, and antistigma campaigns. By enhancing caregivers’ mental well-being and equipping them with the necessary skills and resources, such strategies can sustainably reduce caregiver burden and improve the overall quality of care for individuals with schizophrenia, ultimately contributing to a reduced risk of relapse.

### Limitations

This study had several limitations that should be considered. First, the cross-sectional design limits the ability to draw causal conclusions between variables. As the data were collected at only one point in time, the dynamics of caregiver stress and its impact on long-term well-being cannot be fully explored. Therefore, longitudinal studies are recommended to observe changes in caregivers’ conditions over time. Additionally, the sample was limited to female caregivers from a specific geographic region, which may have affected the generalizability of the findings. Future research should involve male caregivers and explore cultural differences in their stress responses.

### Comparison With Prior Work

These findings align with the current literature indicating that caregivers often face emotional strain, high caregiving burdens, and limited social support and digital health literacy, especially in resource-limited settings [47-49].

### Level Stress Caregivers

The majority of caregivers (58/110, 53%) reported experiencing mild stress, whereas 5% (6/110) reported severe stress. These findings support previous literature suggesting that caregiver stress exists on a spectrum and is influenced by factors such as caregiving duration, social support, and coping capacity [50,51]. The predominance of mild stress suggests the potential for resilience, particularly within collective cultures such as Indonesia, where family roles are highly valued [52].

Caregivers with mild stress experience ongoing psychological pressure related to their caregiving responsibilities. Caregivers typically have strong social support and functional coping mechanisms [46,53]. In contrast, caregivers who experience severe stress are more vulnerable to emotional exhaustion, social isolation, and stigma associated with mental illness. These factors align with the Transactional Model of Stress and Coping [40], which posits that stress arises when caregiving demands exceed an individual’s coping ability.

These findings underscore the importance of social buffering, whereby social support mitigates the negative effects of stress. Family and community support has been shown to significantly reduce caregiver stress [46,54,55]. From a practical standpoint, these findings highlight the need for routine screening for caregiver stress as part of a holistic and preventive health care approach. Digital interventions, such as digital psychoeducation and caregiver support apps, have proven effective in improving coping skills and reducing stress [40,46,53-55]. By understanding the distribution and determinants of caregiver stress, health care providers can design more comprehensive, sustainable, community-based, and technology-driven intervention strategies tailored to local social and cultural contexts. Such strategies can enhance caregivers’ quality of life and help prevent relapses in individuals with schizophrenia.

### Significant Determinant Factors of Caregiver Stress

Results revealed that several personal and psychosocial variables were significantly associated with caregiver stress. Demographic characteristics, such as age (particularly those aged 45‐55 y) and lower levels of education, were significantly related to increased stress. These findings indicate the importance of tailoring interventions based on age-related needs and cognitive capacity. Older caregivers with limited formal education may face challenges in navigating digital platforms or comprehending complex medical information, which hinders their ability to effectively manage caregiving responsibilities. Previous studies have shown that web-based psychoeducational interventions can bridge this gap. For instance, Voineskos et al [56] found that tailored digital modules improved caregiver understanding and stress management, particularly among low-educated groups. Similarly, Mueser et al [57] emphasized that user-friendly interfaces and multimedia content significantly enhance engagement among digitally inexperienced caregivers. Beyond education and age, caregiver burden was strongly associated with higher stress levels (*P*<.001), consistent with prior findings that caregiving over extended periods, lack of respite, and insufficient social support contribute to both physical and psychological fatigue [58].

Coping strategies also showed a significant association (*P*<.001), with maladaptive strategies such as avoidance and denial being linked to elevated stress levels, while adaptive coping approaches such as peer support were associated with better psychological outcomes [59,60]. Moreover, caregiver knowledge played a pivotal role (*P*<.001). Caregivers with higher levels of illness literacy were better equipped to manage care challenges, align expectations, and support adherence to treatment. As explained by Al-Awad [61], when caregivers understand the rationale behind treatment protocols, medication adherence improves, although stigma and limited accessibility can obstruct compliance. Notably, medication nonadherence (*P*=.004) was significantly associated with stress, reinforcing the need for education-based strategies. These findings align with digital health research [62], which showed that mobile coping programs enhanced caregivers’ health literacy and emotional regulation, thereby reducing stress. Another critical factor was stigma (*P*=.003). As framed by Goffman’s Social Stigma Theory, mental illness-related stigma can lead to social exclusion and emotional strain in caregivers. Shimazu [63] supported this finding, noting that caregivers of people with schizophrenia often internalize stigma, exacerbating emotional distress.

In contrast, social support (*P*=.26) and access to health information (*P*=.25) were not statistically significant in this study, although their relevance was supported by existing theory. According to the Social Support Theory, emotional and instrumental support buffers individuals from stress. Fonseka and Woo [64] emphasized that even when caregivers receive support, those who perceive it as insufficient remain at psychological risk [65]. Similarly, while information access did not show a statistically significant relationship with stress, its theoretical importance remained. As proposed by the Health Information Access Theory, equitable access to health information enhances health literacy, decision-making, and caregiving confidence. Castillo et al [46] and Romm et al [65] found that disparities in access could hinder caregivers’ coping mechanisms and increase anxiety. Chen et al [66] also found that uncertainty and poor access to accurate information exacerbated emotional burdens among caregivers [67].

These findings reinforce the notion that caregiver stress is shaped by a complex interplay of personal, social, and informational variables. Interventions should be designed holistically, addressing not only empirically significant variables such as education, burden, and stigma but also theoretically important elements such as social support and information equity to foster comprehensive caregiver resilience and reduce psychological strain. Thus, these findings not only align with but also extend existing theoretical frameworks by offering practical implications for designing targeted, evidence-based interventions that address both individual and systemic determinants of caregiver stress.

Structural equation modeling using PLS-SEM confirmed that caregiving burden, knowledge, social support, and stigma were significant predictors of caregiver stress. The model demonstrated good internal consistency (Cronbach α>0.70), adequate convergent validity (AVE>0.50), and strong outer loading (>0.60). The PLS-predict analysis further supported the model’s predictive relevance, outperforming benchmark linear regression models. These findings underscore the need for culturally contextualized, targeted interventions, particularly digital health solutions that address psychosocial stressors and improve caregiver literacy and resilience in mental health care.

### Conclusions

This study revealed that the majority of caregivers of individuals with schizophrenia experienced mild stress; however, a substantial subset also encountered moderate to severe stress. Key psychosocial determinants included caregiving burden, stigma, insufficient knowledge, and limited social support. These findings provide important empirical evidence that emphasizes the urgency of developing targeted support strategies for caregivers.

Given the growing role of digital health in improving mental health outcomes, future research should focus on the design and evaluation of telehealth-based interventions. Specifically, randomized controlled trials and longitudinal studies are required to assess the long-term effectiveness of community-based psychoeducational programs delivered through mobile or web platforms. These interventions should be tailored to caregivers’ digital and health literacy levels, particularly in resource-constrained settings, to ensure accessibility and sustained engagement.

## Supplementary material

10.2196/70648 Multimedia Appendix 1 Data analysis model of partial least squares structural equation modeling (PLS-SEM).

10.2196/70648 Checklist 1 Checklist for Reporting Results of Internet E-Surveys (CHERRIES)

## Abbreviations

AVE: average variance extracted
CHERRIES: Checklist for Reporting Results of Internet E-Surveys
PLS: partial least squares
SEM: structural equation modeling
