# Supplementary material for: Determinant Factors of Stress in Caregivers of Patients With Schizophrenia: Cross-Sectional Study
Source: JMIR Form Res. 2025 Jul 3;9:e70648. doi: 10.2196/70648 (PMC12244270; doi:10.2196/70648)
Supplement: Multimedia Appendix 1 [file formative-v9-e70648-s001.pdf]

Data analysis model SEM PLS

Table S1. Validity and reliability of Assessment Tools

Assessment tools	Realibility
National Alliance for Caregiving Questionnaire in collaboration with AARP	0.982 [a]
Internalized Stigma of Mental Illness (ISMI) scale	0.9 [b]
the *COPE Inventory* and the *McMaster Family Assessment Device (FAD)*	0.989 [b]
Multidimensional Perceived Social Support (MPSS)	0.952 [a]
Zarit's Caregiver Burden	0.989 [b]
Knowledge Assessment Schizophrenia Test (KAST)	0.966 [b]
Medication Compliance Report Scale (MARS)	0.949 [b]

a : p< 0.01, b : p< 0.000,

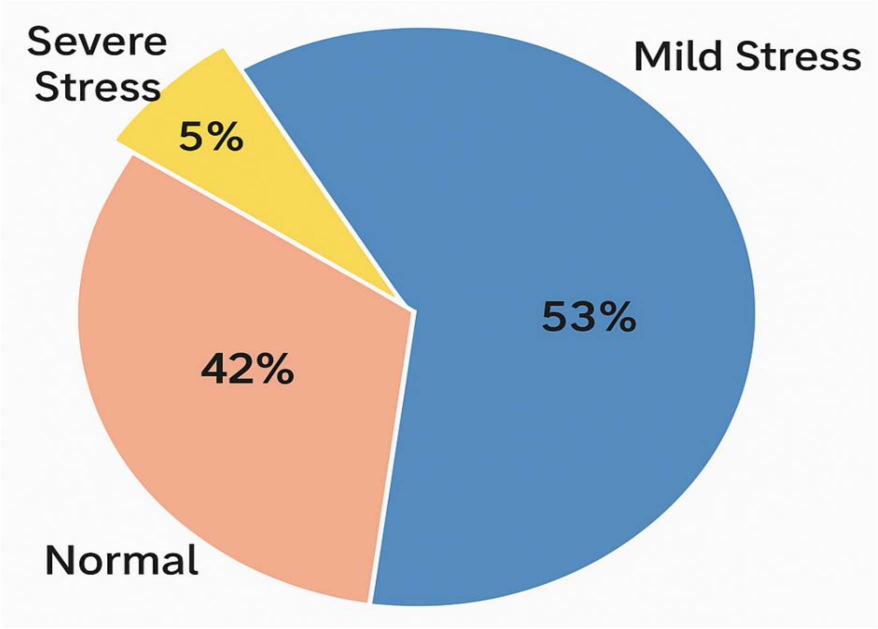

Figure S1. Distribution Level Caregiver of Schizophrenia Patients in Pangkep Regency, Indonesia

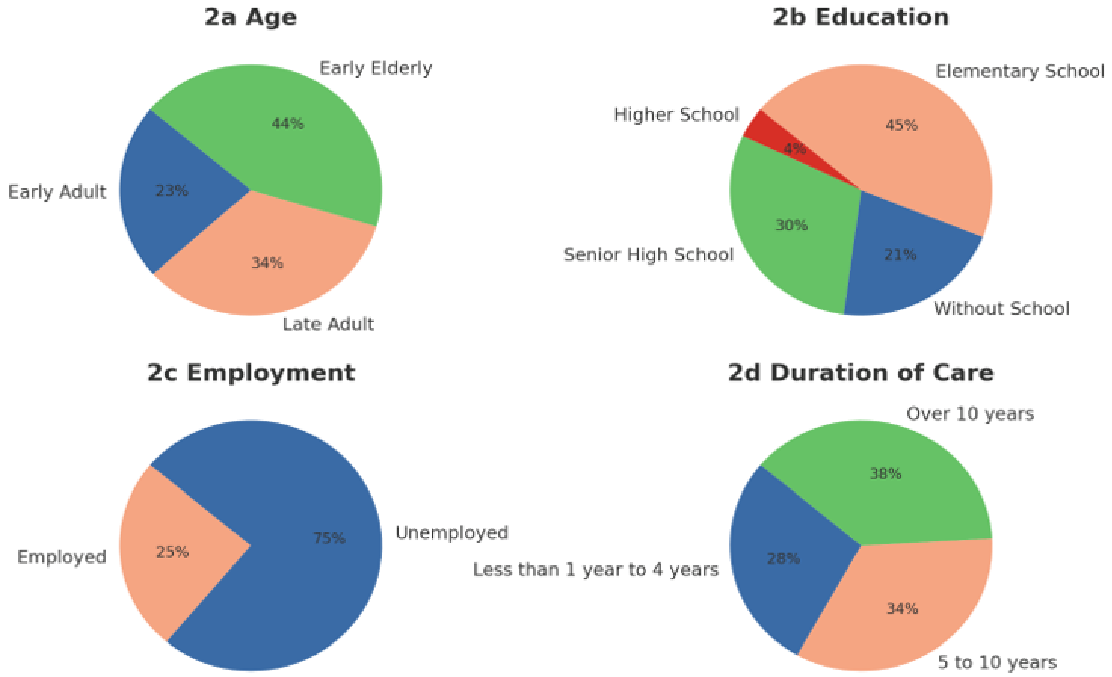

Figure S2. Distribution of Personal Dterminant Factors of Schizophrenia Patients in Pangkep Regency, Indonesia

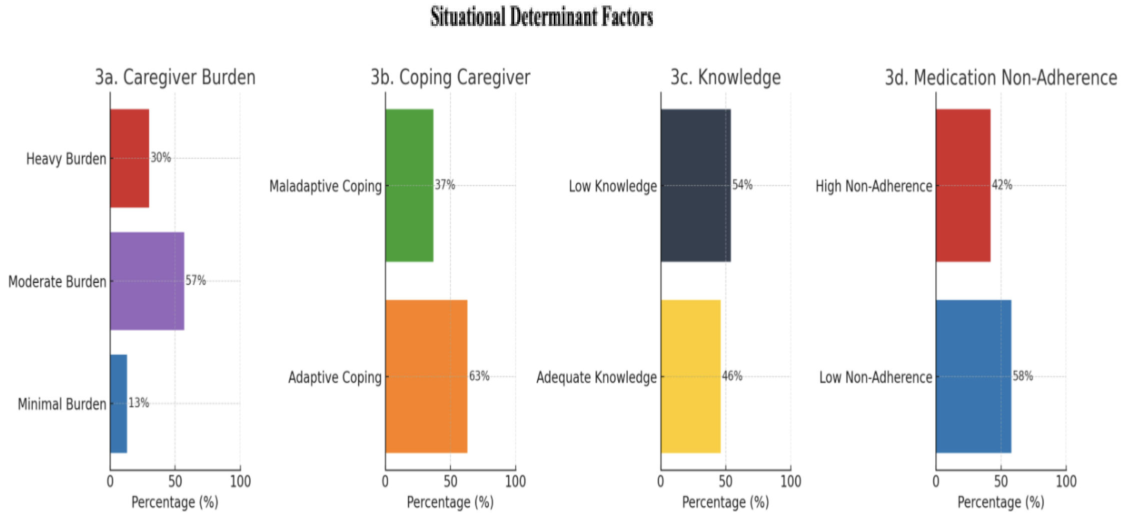

Figure S3. Distribution of Situational Determinant Factors of Schizophrenia Patients in Pangkep Regency, Indonesia

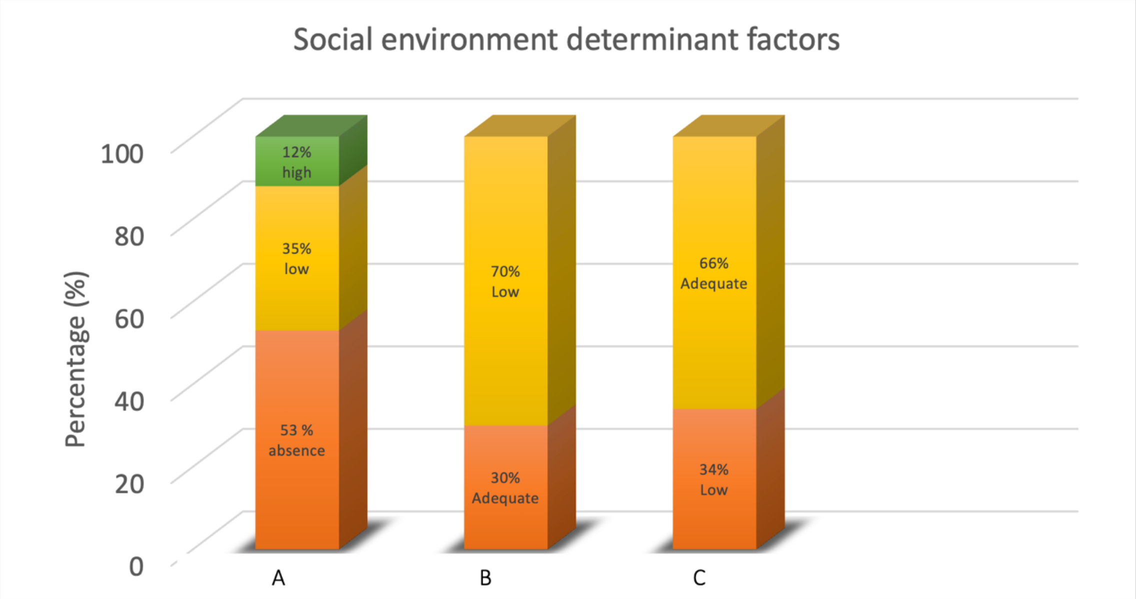

Figure S4. Distribution of Social Environment Determinant Factors of Schizophrenia Patients in Pangkep Regency, Indonesia

Table S2. Determinant Factors of Stress Level on Caregivers of Schizophrenia Patients in Pangkep Regency, Indonesia, Cross-Sectional Study, 2024

Personal Determinant Factors	Stress Level						Total		p[a]
	Normal		Mild Stress		Severe Stress				
	n	%	n	%	n	%	n	%	
**Age**									
Early Adult	8	7.3	15	13.7	2	1.8	25	22.7	0.030[a]
Late Adult	16	14.5	19	17.3	2	1.8	37	33.6	
Early Elderly	22	20.0	24	21.8	2	1.8	48	43.7	
**Education**									
Without School	12	10.9	11	10.0	0	0.0	23	20.9	<.001[ab]
Elementary School	28	25.5	10	9.1	12	10.9	50	45.5	
Senior High School	6	5.5	26	23.7	1	0.9	33	30.0	
Higher School	0	0.0	4	3.6	0	0.0	4	3.6	
**Employment**									
Employed	16	14.5	10	9.1	1	0.9	27	24.5	0.053
Unemployed	30	27.3	48	43.6	5	4.5	83	75.5	
**Duration of Caregiving**									
< 1 year to 4 years	18	16.4	13	11.7	0	0.0	31	28.2	0.075
5 to 10 years	15	13.6	21	19.1	1	0.9	37	33.6	
Over 10 years	13	11.8	24	16.4	5	4.5	42	38.2	
**Burden Caregiver**									

Minimal	13	11.8	1	0.9	0	0.0	14	12.7	<.001[ab]
Moderat Burden	32	29.1	25	24.5	4	3.6	63	57.3	
High Burden	1	0.9	30	27.2	2	1.8	33	30.0	
**Coping caregiver**									
Adaptif	10	9.1	54	49.1	6	5.5	69	62.7	<.001[ab]
Maladaptif	36	32.7	4	3.6	1	0.9	41	37.3	
**Knowledge**									
Adequate	45	40.9	6	5.4	0	0.0	51	46.7	<.001 [ab]
Low	1	0.9	36	32.7	6	5.5	59	53.6	
**Medication non-compliance**									
High	11	10.0	34	31.0	1	0.9	46	41.8	0.004[a]
Low	35	31.8	24	21.9	5	4.5	64	58.2	
**Stigma**									
Absence	12	10.9	26	23.7	1	0.9	39	35.5	0.003[a]
Low	34	30.9	29	26.3	5	4.5	70	64.7	
High	0	0.0	3	2.7	0	0.0	3	2.7	
**Social Support caregiver**									
Adequate	34	30.9	38	34.5	5	4.5	77	70.0	0.263
Low	12	10.9	20	18.1	1	0.9	33	30.0	
**Access to Health Information**									
Adequate	13	11.8	23	20.9	1	0.9	37	33.6	0.254
Low	33	30.0	35	31.9	5	4.5	73	66.4	

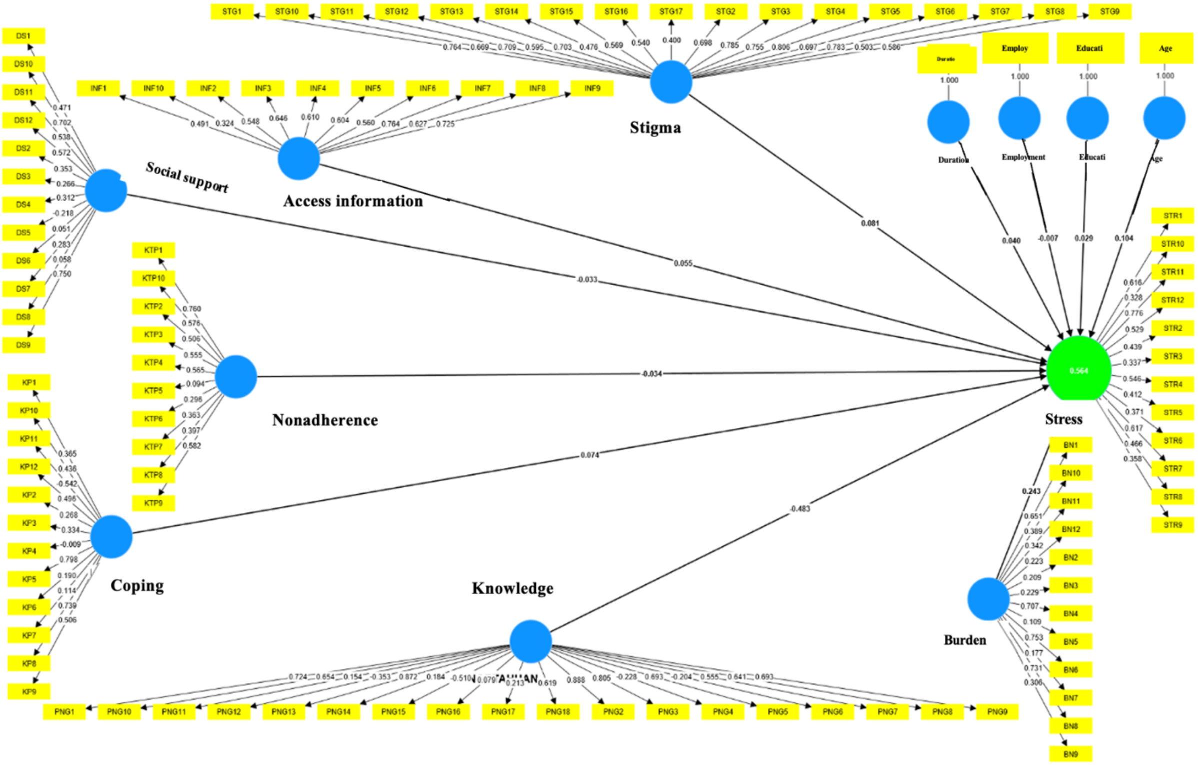

Figure S5. PLS Model Specification Diagram (First Estimation)

Table S3. Reliability Level of Re-specification (First Estimation) Determinant Factors of Stress Level on Caregiver of Schizophrenia Patients in Pangkep Regency, Indonesia, Cross-Sectional Study, 2024

Dimension	Cronbach's alpha[a]	Composite reliability (rho_a)[b]	Composite reliability (rho_c)[c]	Note
Access to health Information	0.842	0.829	0.845	Reliable
Social Support Cargiver	0.622	0.677	0.639	Reliable
Stigma	0.918	0.934	0.927	Reliable
Burden cargiver	0.709	0.721	0.712	Reliable
Medication Non Adherence	0.677	0.685	0.746	Reliable
Coping cargiver	0.622	0.564	0.591	No Reliable
Knowledge	0.685	0.916	0.775	Reliable
Stress	0.715	0.757	0.789	Reliable

[a] alpha < 0.6 Non Realibel, [b] rho_a (compsoite reliability) >0.6, [*]Realibel if [a] alpha and [b] rho_a > 0.6

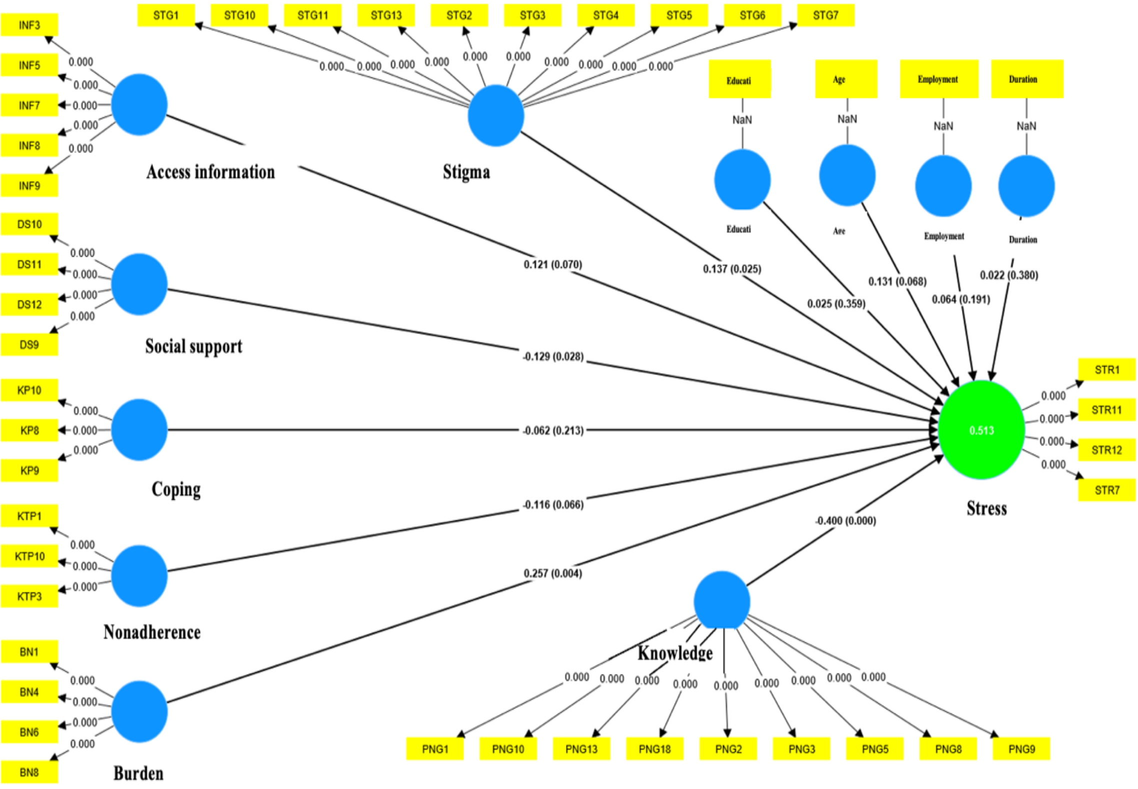

Figure S6. Outer Model Diagram of PLS Re-specification (Second Estimation)

Table S4. Reliability Level of Second Estimation on Determinant Factors of Stress Level on Caregiver of Schizophrenia Patients in Pangkep Regency, Indonesia, Cross Sectional Study, 2024

Dimension	Cronbach's alpha [a]	Composite reliability (rho_a) [b]	Composite reliability (rho_c) [c]	Average variance extracted (AVE) [d]
Access to health information	0,561	0,705	0,807	0,680
Social support caregiver	0,806	0,872	0,887	0,727
Burden Cargiver	0,659	0,672	0,815	0,597
Stigma	0,916	0,921	0,930	0,548
Medication Non Adherence	0,775	0,820	0,897	0,813
Coping Caregiver	0,737	0,790	0,849	0,655
Knowledge	0,897	0,909	0,918	0,588
Stress	0,710	0,728	0,821	0,537

[a] alpha < 0.6 Non Realibel, [b] **rho_a** (composite reliability) > 0.6, [c] **rho_c** (composite reliability) > 0.6, [d] **AVE** > 0.6, *Realibel if [a] alpha and [b] **rho_a** > 0, **model PLS accepted if [a] alpha, [b,c]**rho_a** > 0.6, AVE > 0,5

Table S5. PLS Predict

	Q^2predict	PLS-SEM_RMSE	PLS-SEM_MAE	LM_RMSE	LM_MAE
STR1	0,254	0,871	0,640	1,150	0,900
STR7	0,083	0,844	0,597	1,107	0,823

STR11	0,276	0,779	0,579	0,904	0,732
STR12	0,119	1,041	0,812	1,431	1,103
