# Supplementary material for: Determinant Factors of Stress in Caregivers of Patients With Schizophrenia: Cross-Sectional Study
Source: JMIR Form Res. 2025 Jul 3;9:e70648. doi: 10.2196/70648 (PMC12244270; doi:10.2196/70648)
Supplement: Checklist 1 [file formative-v9-e70648-s002.docx]

**Checklist for Reporting Results of Internet E-Surveys (CHERRIES)**

| **Item category** | **Checklist item** | **Your Study** |
| --- | --- | --- |
| **Design** | Describe survey design | Cross-sectional study (N=110). Target population was female caregivers of individuals with schizophrenia in Labakkang District, South Sulawesi, Indonesia. Survey was administered in person using structured and validated paper-based questionnaires. |
| **IRB approval and informed consent process** | IRB approval | This study was approved by the Health Research Ethics Committee of [Hasanuddin Unisersity], approval number [25240930270]. |
|  | Informed consent | Written informed consent was obtained from all participants after explaining the study’s purpose, procedures, and confidentiality safeguards. |
| **Data protection** | The survey did not include identifiable information; participants were anonymized. |  |
| **Development and pre-testing** | Questionnaire development | The questionnaire was developed based on existing literature and expert input, and included validated instruments to assess stress levels, caregiver burden, coping, knowledge, stigma, and social support. |
|  | Testing | Pilot testing was conducted with 10 caregivers to assess clarity and relevance, leading to minor refinements. |
| **Recruitment process and sample** | Open survey vs closed survey | Closed survey. Only pre-identified caregivers attending the health center were approached. |
|  | Contact mode | Caregivers were contacted and recruited directly through the Labakkang District Health Center. |
|  | Advertising the survey | Not applicable. |
| **Survey administration** | Web/email | Paper-based self-administered questionnaire. |
|  | Context | Conducted in a clinical community health setting. |
|  | Mandatory/voluntary | Participation was voluntary. |
|  | Incentives | No incentives were provided. |
|  | Time/Date | Data were collected from June to August 2024. |
| **Randomization of items** | Items or questionnaires not randomized. |  |
| **Adaptive questioning** | Adaptive questioning not applied. |  |
| **Number of items** | The questionnaire contained 60 items. |  |
| **Number of screens/pages** | Not applicable (paper-based format). |  |
| **Completeness check** | Responses were reviewed immediately upon submission; incomplete responses were excluded. |  |
| **Review step** | Participants could revise their responses before final submission. |  |
| **Response rates** | Unique site visitor | Not applicable. |
|  | View rate | Not applicable. |
|  | Participation rate | Not applicable. |
|  | Completion rate | All 110 distributed questionnaires were completed and included in the analysis. |
| **Preventing multiple entries** | Cookies used | Not applicable. |
|  | IP check | Not applicable. |
|  | Log file analysis | Not applicable. |
|  | Registration | Participants were identified through health records and invited once. |
| **Analysis** | Handling incomplete questionnaires | Incomplete questionnaires were excluded. |
|  | Atypical timestamps | Not applicable. |
|  | Statistical correction | Statistical analysis included chi-square tests and PLS-SEM modeling using SmartPLS to identify determinant factors of stress. |
